# Acute saddle pulmonary embolism on ^18^F‐FDG PET/CT: diagnosis by functional imaging

**DOI:** 10.1002/rcr2.476

**Published:** 2019-08-20

**Authors:** Dalveer Singh, Roslyn Foessel, Navjot Nagra, Pauline Lau, Damian Brauchli

**Affiliations:** ^1^ Department of Nuclear Medicine Qscan Radiology Clinics Brisbane Australia; ^2^ School of Medicine University of Queensland Brisbane Australia

**Keywords:** Positron emission tomography/computed tomography, pulmonary embolus, right heart strain, rim sign

## Abstract

^18^F‐labelled fluoro‐2‐deoxyglucose positron emission tomography/computed tomography (^18^F‐FDG PET/CT) is used extensively in the setting of cancer staging and in assessing cancer treatment response. Oncology patients have a sevenfold risk of developing pulmonary embolism (PE) due to underlying activation of the haemostatic system and anti‐cancer therapy inducing a hypercoagulable state. The diagnosis of PE on ^18^F‐FDG PET/CT is challenging, particularly in the absence of intravenous contrast. The case of a female patient undergoing treatment for advanced diffuse large B‐cell lymphoma is presented. The ancillary signs of PE are illustrated on consecutive non‐contrast‐enhanced ^18^F‐FDG PET/CT scans. The signs include the “rim sign” relating to regions of pulmonary infarction and abnormal cardiac uptake indicating right heart strain. The diagnosis was confirmed on CT pulmonary angiography which demonstrated extensive PE, including a saddle embolus.

## Introduction

Pulmonary embolism (PE) is the second leading cause of death in patients with cancer 1. Overall, the risk of PE is sevenfold in oncology patients compared with the general population 2, which is attributable to inherent cancer‐mediated mechanisms and anti‐cancer therapies inducing a hypercoagulable state. Cancers that are at particularly high risk are haematological, lung, and gastrointestinal (28‐fold, 22‐fold, and 20‐fold risk, respectively). Coincidentally, ^18^F‐labelled fluoro‐2‐deoxyglucose positron emission tomography/computed tomography (^18^F‐FDG PET/CT) is an extremely useful tool in this subset of cancers and is performed in the majority of patients for staging and to assess treatment response. In the setting of non‐contrast‐enhanced ^18^F‐FDG PET/CT, it is important to recognize the indirect signs of PE so that patients can be appropriately treated. We present a case illustrating typical non‐contrast‐enhanced ^18^F‐FDG PET/CT findings of PE in a patient undergoing treatment for diffuse large B cell lymphoma (DLBCL).

## Case Report

**Figure 1:**
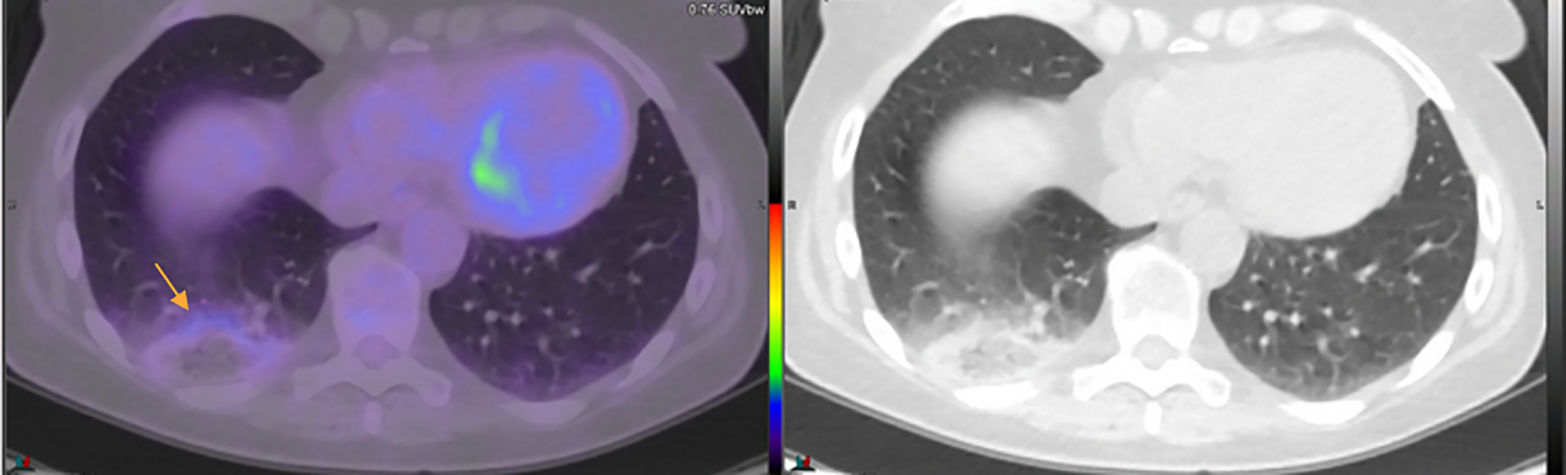
Right lower lobe opacity demonstrating the “rim sign” (arrow).

A middle‐aged female presented for a non‐contrast ^18^F‐FDG PET/CT scan for restaging of advanced DLBCL post completion of R‐CHOP (rituximab cyclophosphamide doxorubicin) chemotherapy. She had known widespread nodal and extra‐nodal disease (including brain), and documented episodes of acute tachycardia and dyspnoea seven weeks earlier. The non‐contrast CT images demonstrated a peripheral area of ground glass with surrounding consolidation in the posterior right lower lobe. The PET data confirmed a corresponding rim of low‐level ^18^F‐FDG uptake with central photopaenia, consistent with the “rim sign” (Fig. 1).

**Figure 2:**
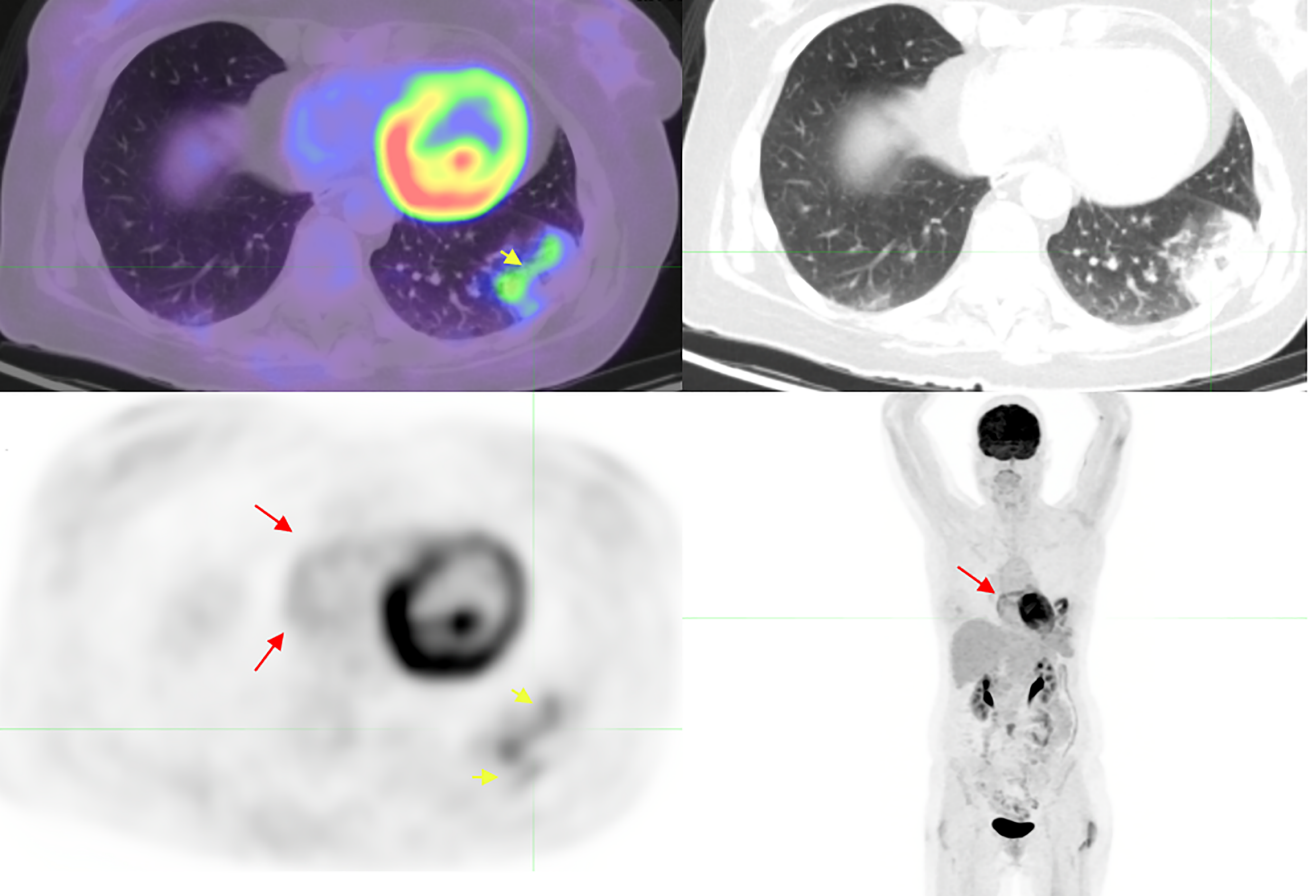
Left lower lobe “rim sign” (short arrow) and four‐chamber cardiac uptake (best appreciated on the whole body maximal intensity projection (long arrow).

Four weeks later while the patient was undergoing whole brain radiation for residual intracranial DLBCL, the patient returned for an ^18^F‐FDG PET/CT surveillance scan. Peripheral ground glass with a rim of low‐level ^18^F‐FDG uptake was again demonstrated, this time within the left lower lobe (Fig. 2) with the additional finding of four‐chamber cardiac ^18^F‐FDG uptake.

The patient deteriorated with acute tachycardia, dyspnoea, and left pleuritic chest pain three hours after the scan. A CT pulmonary angiography (CTPA) was performed, diagnosing extensive lobar, segmental, subsegmental, and saddle PE. There were no features of right heart strain (RHS) on the CTPA.

After four weeks of oral anticoagulants and completion of therapy for DLBCL, a follow‐up ^18^F‐FDG PET/CT scan showed complete resolution of the pulmonary changes and complete metabolic response to therapy.

## Discussion

Incidental PE was found in 0.32% of contrast‐enhanced ^18^F‐FDG PET/CT scans in one retrospective study 3. Protocolling for ^18^F‐FDG PET/CT depends on practice‐specific preferences and patient contraindications such as renal impairment and prior contrast reaction. Many patients will undergo a non‐contrast study which poses even greater challenge for diagnosing PE. Nonetheless, the importance of recognizing potential PE cannot be overstated given the high pre‐test probability in the at‐risk population of oncology patients and the potential for catastrophic outcomes in undiagnosed PE. In these patients there is a reliance on secondary signs of PE. In addition, where contrast is used it is typically acquired with contrast in an arterial phase and during free respiration, which is far from ideal when attempting to detect small and/or basal emboli. Therefore, even when contrast‐enhanced ^18^F‐FDG PET/CT is used, an understanding of the indirect signs of PE is invaluable.

The two most distinguishable signs of a PE on a non‐contrast‐enhanced ^18^F‐FDG PET/CT scan are the “rim sign” which indicates a pulmonary infarction and abnormal cardiac uptake associated with RHS. Other signs include hypermetabolism and hypometabolic filling defects in the pulmonary artery, but these are less reliable. A pulmonary infarction manifests as an area of geographical coagulative necrosis in the subpleural region with islands of viable lung parenchyma and a reparative margin of granulation tissue with a well‐defined edge consisting of palisading histiocytes and foamy macrophages 4. The peripheral inflammation demonstrates variable FDG uptake resulting in the “rim sign.” As pulmonary infarctions are absent in the majority of cases of PE, the absence of “rim sign” in no way reliably excludes the diagnosis 5. Additionally, a centrally necrotic tumour can mimic the appearance of the “rim sign” and the degree of uptake similar to low‐grade primary lung neoplasm such as adenocarcinoma. In our case, observing the pulmonary infarct on CT may have prompted search for PE via CTPA earlier; however, this requires astute interpretation and high index of suspicion.

The presence of alternative indirect signs for PE such as RHS in this case report can greatly increase diagnostic confidence. Under normal physiological circumstances, only the left ventricle will typically show FDG uptake. In the setting of PE, increased pulmonary arterial resistance and tachycardia contribute to increased metabolic demand, which can alter the distribution of FDG within the heart. Uptake within the right ventricular wall and both atria has been described 3, 6. It should be noted that RHS is not specific to PE and can also present due to underlying heart or lung disease.

In this case, the patient had moderate right atrial uptake and only very subtle right ventricular uptake. This is a different pattern to the case reported by Franceschi et al. 6 describing intense right atrial and ventricular uptake. In this case, the patient was asymptomatic at the time of the scan and subsequently deteriorated. This raises the possibility that right atrial uptake is an earlier sign of RHS than right ventricular uptake, although this would require further study to confirm. Indeed observing a new finding of RHS should prompt the interpreting physician to enquire to the clinical status of the patient, communication with the referring clinician to ascertain the need for urgent CTPA.

PE is a challenging diagnosis on non‐contrast‐enhanced ^18^F‐FDG PET/CT. While many cases of PE will remain undetectable, awareness of indirect signs such as the “rim sign” and abnormal cardiac FDG uptake may prevent potentially catastrophic outcomes in at‐risk oncology patients.

### Disclosure Statement

Appropriate written informed consent was obtained for publication of this case report and accompanying images.
